# Correlation of Naturally Occurring HIV-1 Resistance to DEB025 with Capsid Amino Acid Polymorphisms

**DOI:** 10.3390/v5030981

**Published:** 2013-03-22

**Authors:** Philippe A. Gallay, Roger G. Ptak, Michael D. Bobardt, Jean-Maurice Dumont, Grégoire Vuagniaux, Brigitte Rosenwirth

**Affiliations:** 1 Department of Immunology & Microbial Science, The Scripps Research Institute, La Jolla 92037, California, USA; E-Mails: gallay@scripps.edu (P.A.G.); mbobardt@scripps.edu (M.D.B.); 2 Southern Research Institute, Frederick, Maryland 21701, USA; E-Mail: ptak@southernresearch.org; 3 Debiopharm, 1002 Lausanne, Switzerland; E-Mail: gvuagniaux@debiopharm.com; 4 Klinisches Institut fuer Virologie, Medizinische Universitaet Wien, 1095 Vienna, Austria; E-Mail: brigitte.rosenwirth@inode.at

**Keywords:** DEB025, alisporivir, cyclophilin inhibitors, cyclosporines, HIV/retroviruses, HIV capsid, natural resistance

## Abstract

DEB025 (alisporivir) is a synthetic cyclosporine with inhibitory activity against human immunodeficiency virus type-1 (HIV-1) and hepatitis C virus (HCV). It binds to cyclophilin A (CypA) and blocks essential functions of CypA in the viral replication cycles of both viruses. DEB025 inhibits clinical HIV-1 isolates *in vitro* and decreases HIV-1 virus load in the majority of patients. HIV-1 isolates being naturally resistant to DEB025 have been detected *in vitro* and in nonresponder patients. By sequence analysis of their capsid protein (CA) region, two amino acid polymorphisms that correlated with DEB025 resistance were identified: H87Q and I91N, both located in the CypA-binding loop of the CA protein of HIV-1. The H87Q change was by far more abundant than I91N. Additional polymorphisms in the CypA-binding loop (positions 86, 91 and 96), as well as in the N-terminal loop of CA were detected in resistant isolates and are assumed to contribute to the degree of resistance. These amino acid changes may modulate the conformation of the CypA-binding loop of CA in such a way that binding and/or isomerase function of CypA are no longer necessary for virus replication. The resistant HIV-1 isolates thus are CypA-independent.

## 1. Introduction

Cyclophilins are ubiquitous cellular proteins that possess *cis*-trans prolyl isomerase (PPIase) activity and are believed to act in protein folding and as chaperones in intracellular transport [1,2]. Cyclophilin A (CypA), a cytosolic member of this family of proteins, is involved in the replication of human immunodeficiency virus type 1 (HIV-1) [3,4,5]. CypA is also a crucial cellular cofactor for hepatitis C virus (HCV) replication [6,7,8,9]. CypA fulfills an essential function early in the HIV-1 lifecycle. It binds specifically to a proline-containing loop formed by amino acids 85–93 of capsid protein p24 (CA) [10,11,12]. The step in the viral replication cycle, where CypA is involved, is an event after penetration of HIV-1 and before integration of the double-stranded viral DNA into the cellular genome, often referred to as “uncoating” [13,14,15]. In HCV replication, CypA is assumed to act as a stimulatory regulator of NS5A in the viral RNA synthesis machinery [6,7,8,9,16,17].

Cyclophilins were originally identified as intracellular receptor molecules for cyclosporines, a class of cyclic undecapeptides produced by the fungus, *Tolypocladium inflatum* [18,19,20]. The most prominent representative of this class of compounds is cyclosporine A (CsA), an inhibitor of T-cell activation, which is used in the clinic as an immunosuppressant in organ transplantation [21]. CsA, when bound to CypA, forms a ternary complex with calcineurin and, thus, blocks the phosphatase activity of calcineurin that is crucial for activation of T-cells [22,23]. Binding of cyclosporines to cyclophilins also inhibits the *cis*-trans prolyl isomerase (PPIase) activity of these proteins. Two separate domains of CsA can be distinguished that are involved in binding to CypA and calcineurin, respectively [24,25,26,27]. Thus, the immunosuppressive capacity of CsA can be separated from its affinity to CypA by chemical modification.

CypA was identified in 1993 as a target in HIV-1 chemotherapy, when it was discovered that HIV-1 CA protein binds to CypA [4,12]. Inhibition of HIV-1 CA protein interaction with CypA, by competitive binding of cyclosporines to CypA, effectively blocked HIV-1 replication [14,28]. Evaluation of various cyclosporines against HIV-1 revealed that the immunosuppressive capacity was not needed for virus inhibition [13,29]. However, a clear correlation between antiviral activity and binding affinity of cyclosporines to CypA and, thus, their isomerase inhibiting potency, was evident. (Methyl-Ile4)cyclosporine (NIM811), a non-immunosuppressive cyclosporine with high binding affinity for CypA and potent isomerase inhibiting activity, was characterized in detail for inhibition of HIV-1 [5,13,15,28,30,31,32,33] and, more recently, as an inhibitor of HCV replication [30,31,32,34]. NIM811 was evaluated in a clinical pilot study in combination with interferon in chronically HCV-infected patients [35].

**Figure 1:**
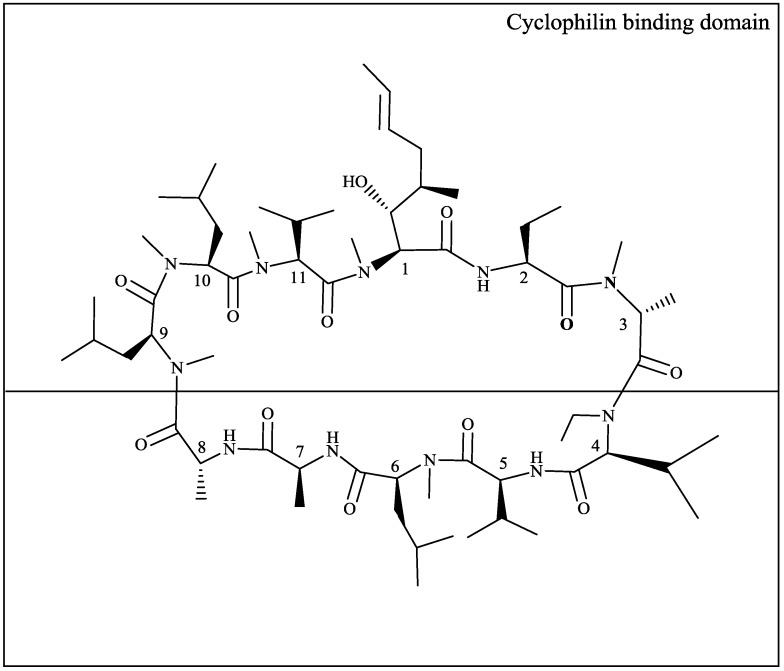
Structural formula of DEB025. The top part of the structure represents the cyclophilin-binding domain.

Based on the knowledge about structure-activity relationships regarding immunosuppressive capacity, CypA-binding affinity and anti-HIV-1 activity [13,23,36,37], (D-Methyl-Ala3-Ethyl-Val4) cyclosporine, (DEB025, alisporivir), was synthesized by chemical modification of CsA [38]. The structural changes in positions 3 and 4 conferred to DEB025 increased CypA binding/isomerase inhibiting capacity (determinant for antiviral activity) and abolished calcineurin affinity (responsible for immunosuppression) [39,40,41] (Figure 1). NMR data and molecular modeling confirmed that DEB025 optimally interacts with CypA and that the interaction of the CypA-DEB025 complex with calcineurin is impeded by steric hindrance due to the Val4 residue [42]. DEB025 proved superior to CsA and NIM811 in inhibiting CypA-associated isomerase activity, as well as in blocking HIV-1 replication [41,43,44]. Mode of action studies proved that DEB025—as other cyclosporines—competitively inhibited interaction of CypA with the CA protein of HIV-1. The point of attack of DEB025 was early in the viral replication cycle: progression/completion of reverse transcription and, consequently, nuclear import of viral DNA were impaired [41,43,44]. The higher affinity of DEB025 to CypA also translated into improved activity against HCV as compared to CsA and NIM811 [40,45,46]. Lack of immunosuppressive effects of DEB025 compared to those of CsA was demonstrated *in vitro* and *in vivo* [41].

DEB025 selectively and potently inhibited replication of laboratory strains and clinical isolates of various HIV-1 subtypes in cell lines and in primary cells [41]. Several isolates being naturally resistant to DEB025 were detected. CA protein from these viruses still bound CypA; however, viral replication was not inhibited by blocking this CA-CypA interaction by DEB025 [43]. Thus, these HIV-1 isolates are CypA independent for their replication. Results showed that naturally occurring capsid substitutions can abolish the need for CypA. Here, we extended the evaluation of HIV-1 isolates for inhibition of replication by DEB025 and, by sequence analysis of the CA region of identified naturally resistant strains, we defined amino acid polymorphisms associated with resistance to DEB025.

DEB025 was evaluated in a clinical study in HIV-1/HCV-coinfected patients [47]. DEB025 treatment induced a significant reduction in log10 HCV RNA copies/ml as compared to placebo. The reduction in HCV RNA copies (mean maximum reduction ˗3.63 log10 copies/ml) was considerably larger than the observed decrease in HIV-1 RNA log10 copies/ml (mean maximum reduction was ˗1.03 HIV-1 log10 copies/ml), which was, however, significant as compared to baseline values. Regarding HIV-1, large variation in response to treatment between individual patients was noted: the best response was a decrease of -1.9 HIV-1 RNA log10 copies/ml; however, four out of 19 patients did not respond to DEB025 treatment with a decrease in HIV-1 virus load. The promising activity of DEB025 in reducing HCV virus loads in chronically infected patients was confirmed in further clinical studies [48,49]. DEB025 (Alisporivir) is currently in advanced clinical development (phase III) for chronic HCV infection. Here, we investigated if the lack of response to DEB025 with regard to HIV-1 virus load in some patients was due to natural resistance of these patients’ viruses and if this resistance corresponded to mutations in the CA region. The aim was to define, by sequence analysis, the molecular basis for the natural resistance to DEB025 of certain HIV-1 strains observed *in vitro* and *in vivo*.

## 2. Results and Discussion

### 2.1. HIV-1 CA Amino Acid Polymorphisms Associated with Resistance to DEB025

HIV-1 clinical isolates from subtypes A, B, C, D, AE, F, G and O being naturally resistant to DEB025 inhibition have been described [41]. Sequence analysis of the CypA binding loop of CA from these viruses indicated the importance of the amino acid change H87Q, but other amino acid polymorphisms in positions 86, 91 and 96 also appeared to contribute to resistance. Introduction of mutations in these four positions of CA into the DEB025-sensitive strain NL4-3 indeed conferred resistance to DEB025 to the viral constructs [43,50,51]. 

In a screening of 238 HIV-1 clinical isolates in TZM-b1 cells, 15% of the virus strains were found to be naturally resistant to DEB025 to various degrees. For these presumed resistant clinical isolates, inhibition curves were generated. Moderate to high degrees of natural resistance to DEB025 were observed. Isolates showing IC_50_ values higher than 1µM, and several sensitive HIV-1 strains for comparison were analyzed further by correlating IC_50_ values for DEB025 with sequence information of the full-length CA protein. The total CA sequences are given in the Supplementary Materials. Table 1 summarizes the results obtained with laboratory strains and clinical HIV-1 isolates from different subtypes regarding sensitivity to inhibition by DEB025 in TZM-b1 cells and peripheral blood mononuclear cell (PBMC) together with the corresponding CA sequences of amino acids 71 to 130. This CA region includes the CypA-binding loop around G89 and P90 and the small loop around P122. HIV-1 isolates showing significant natural resistance to DEB025 inhibition were detected for subtypes A, B, C, D and AE at a frequency of 6–11%; for subtype F, one out of three tested strains, and for subtype G, all four tested isolates proved resistant. Comparison of CA sequences from the CypA-binding loop revealed that all resistant virus isolates from subtypes A, B, C, D and F contain the H87Q polymorphism. The one exception, isolate CMU02 from subtype AE, does not show the H87Q polymorphism, but a I91N substitution and, in addition, a V86A change in the CA sequence. The other resistant isolate from subtype AE, CMU08, contains H87Q together with I91V and M96L. Analysis of polymorphisms in the CypA-binding loop based on a search of the Los Alamos database yielded the result that H87Q is a rather common polymorphism (19.7%), and H87P is present in 2.4% of the sequences analyzed. The variant I91N, on the contrary, occurs only in 2.6% and V86A in 3.7% of HIV-1 sequences. In our selection of HIV-1 isolates evaluated *in vitro*, the polymorphism I91N was not found in any other virus strain. The association of the I91N polymorphism with resistance to DEB025 is based on the analysis described and is also supported by results from the analysis of virus from nonresponder patients (see below); site-directed mutagenesis studies have not been performed to further confirm this association. 

**Table 1:** Capsid polymorphisms in HIV-1 isolates and correlation with sensitivity to DEB025.

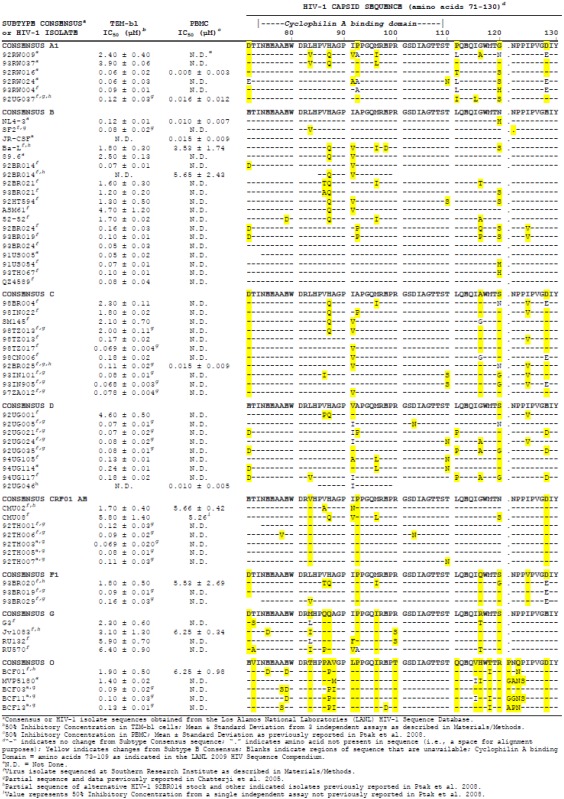

The consensus sequence of subtype G virus strains has the amino acid Q in positions 86 and 87. The sequences of three of the four isolates tested resemble the consensus in these positions; the fourth strain, RU570, shows P in position 86 and Q in 87. Additional amino acid substitutions in positions 91 and 96 of CA are also present. These polymorphisms are assumed to cause the observed resistance to inhibition by DEB025 of all four subtype G isolates tested.

For isolates 92BR014 (subtype B) and 98TZ013 (subtype C), two different virus stocks were tested and sequenced. In both cases, one stock was found to be sensitive, whereas the other was resistant to DEB025 inhibition. The polymorphism H87Q was found in the resistant stocks, but not in the sensitive ones, which confirms the relevance of this amino acid polymorphism.

Other amino acid changes in the CypA-binding loop, like V86A/T/P/Q, I91V/N/L and M96I, have been reported to contribute to resistance [41,43]. Indeed, V86A/T/P/Q is only found in resistant virus isolates; the I91V/L polymorphism, however, occurs in resistant, as well as in sensitive strains, whereas the substitution I91N, as described above, appears to confer resistance to DEB025 in isolate CMU02. The amino acid change M96I, which is only detected in resistant strains, may thus also contribute to resistance in the presence of the H87Q polymorphism. This M96I polymorphism is common to all subtype G strains. 

We did not detect the previously described mutations A92E or G94D [52,53,54] in the sequences from naturally resistant viruses. Variants carrying these changes had been isolated by serial passage of virus strain NL4-3 in CD4^+^ HeLa cells in the presence of the non-immunosuppressive cyclosporine NIM811. These virus variants had shown a phenotype of being resistant or dependent on NIM811 for replication, depending on the cell line used in the assays. However, when grown in primary cells, these mutant viruses were fully sensitive to cyclosporines (Rosenwirth. unpublished; Gallay, unpublished).

### 2.2 Restriction Factors, Cyclophilin A and the Loop around HIV-1 CA Amino Acid P122

In human and nonhuman primate cells, restriction factors confer resistance to infection by retroviruses, blocking an early step in the viral lifecycle. Susceptibility to this inhibition is determined by CA sequences of the retrovirus. In simian cells, the restriction factor TRIM5alpha was identified to mediate the early block to HIV-1 infection. CypA interaction with HIV-1 CA is essential for post-entry inhibition of HIV-1 replication by simian TRIM5alpha [55,56,57,58,59]. On the contrary, CypA is a necessary cofactor for HIV-1 replication in human cells. Blocking CypA-CA interaction by cyclosporines or by certain mutations in the CypA-binding loop of CA, inhibits HIV-1 replication in human cells, but increases it in simian cells. Therefore, it is assumed that CypA-binding to CA protects HIV-1 from an unknown human restriction factor termed Ref-1, while promoting TRIM5alpha mediated restriction in simian cells [60,61,62,63,64,65,66]. Inhibition of retroviral replication by a host cell restriction factor (termed Fv-1) was first described for murine leukemia virus (MLV). The human restriction factor Ref-1 also confers resistance to N-MLV in human cells [61]. MLV CA residue R110, a critical determinant for MLV restriction, aligns with P122 in the CA protein of HIV-1 [67]. The small loop around P122 that includes a mini-beta-hairpin linking CA helices 6 and 7 is sterically in close proximity to the CypA binding loop and makes hydrogen bonding contacts with it [68]. By analogy to MLV, this loop is suspected to play a role in restriction of HIV-1 growth: P122 may be a critical target for Ref-1. If CypA-binding to CA protects HIV-1 from the human restriction factor Ref-1, CypA independence and, thus, resistance to DEB025, could theoretically also be achieved by mutation in the Ref-1 binding region. We found, however, that the small loop around P122 is remarkably conserved in sequence among all clinical isolates tested, except for those of group O (Table 1).

### 2.3. Amino Acid Polymorphisms in CA of HIV-1 Isolates from the Outlier (O) Group

Viruses from group O have been reported to be either dependent or independent of CypA [69,70]. We identified isolates from group O that were either sensitive or resistant to DEB025 inhibition (Table 1), thus confirming the previous results. Remarkably, group O viruses differ from all other subtypes regarding sequences in the CypA-binding loop, as well as in the small loop around P122. Their consensus sequence contains in positions 86, 87 and 88 the amino acids P, A/P and V/M/I, respectively, which differs considerably from the consensus sequences (V, H and A in these positions) of most other group M subtypes. These polymorphisms, however, could not be correlated with sensitivity or resistance to DEB025: the P in positions 86 and 87 occurs in sensitive, as well as in resistant strains of group O. Interestingly, P122 is also replaced by Q or N, and the other residues in this small loop are different for group O viruses as compared to other subtypes, with no obvious correlation to DEB025 resistance. Thus, the independence from CypA of some group O viruses must be due to other not yet identified sequence polymorphisms in CA or the interaction of Ref-1 and/or CypA with CA is different for this group of viruses.

### 2.4. Amino Acid Polymorphisms in CA and *In vitro* Resistance to DEB025 of HIV-1 Isolates from Nonresponder Patients

**Figure 2:**
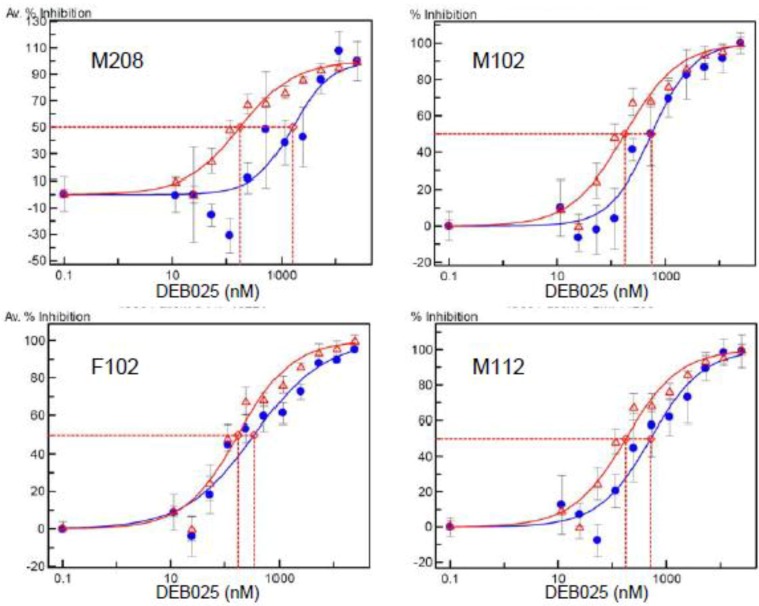
Inhibition curves of recombinant viruses carrying the capsid gene from four patients’ serum specimens. These patients had been nonresponders in a clinical study evaluating DEB025 in HIV-1/HCV-co-infected patients (Flisiak *et al.*, 2008). For each panel, the average percent inhibition effectuated by various concentrations of DEB025 for the recombinant virus (in blue) and the reference virus (strain NL4-3, in red) are depicted. The antiviral activity of DEB025 was determined in the DeCIPhR assay system as described in the Experimental Section.

DEB025 was evaluated *in vitro* (Figure 2) for inhibition of viruses carrying the CA gene of HIV-1 isolates from four patients not responding to DEB025 therapy in a clinical trial [47]. In Table 2, the IC_50_ values for DEB025 are shown, together with the CA sequence of amino acids 71 to 130 from these clinical isolates. The complete CA sequences are given in the Supplementary Materials. All four recombinant viruses exhibit moderate to strong (M208) resistance to DEB025. Two of the viruses (M208 and M112) indeed contain the H87Q polymorphism in their CA sequence and, in addition, V86P, which demonstrates again the importance of these amino acid changes for resistance to DEB025. Remarkably, the virus isolate M102 shows the same I91N polymorphism as the subtype AE strain CMU02 (Table 1), which confirms that this amino acid change can also lead to resistance to DEB025 and to CypA independence of virus replication. The second polymorphism in isolates M102 and M208, M96L, is present in resistant, as well as in sensitive isolates (Table 1). For the virus isolate from nonresponder F102, no amino acid change in CA, except I91V, could be detected; this amino acid change, however, also occurs in sensitive strains (Table 1). This clinical isolate out of the four tested has the lowest IC_50_ value and is, thus, only moderately resistant; it may represent a mixture of sensitive and resistant viruses.

### 2.5. Levels of CypA Expression in PBMCs from Nonresponder Patients

CypA expression levels in different cell lines were reported to modulate HIV-1 replication and sensitivity to cyclosporines [54,71]. To check if different CypA levels may have influenced the clinical response to DEB025, we determined CypA concentrations in the PBMCs of all patients at three time points during the study [47]. These concentrations varied from patient to patient between 0.22µg/mg protein and 1.10µg/mg protein at day-28; however, there was no correlation detectable with clinical response to DEB025. The levels of CypA remained essentially stable within a patient during DEB025 treatment. The PBMC from patients M208, M102, F102 and M112 contained 0.78, 0.60, 0.80 and 0.43 µg CypA per mg protein, respectively, and, thus, were around the mean CypA level (0.54 µg/mg protein).

### 2.6. Amino Acid Polymorphisms outside the CypA Binding Loop

Analysis of the amino acid polymorphisms outside the CypA binding area did not reveal further changes conferring DEB025 resistance in absence of H87Q (see the Supplementary Materials). Two amino acid substitutions in the N-terminal loop of CA, namely L6M and I15M, occur only in resistant strains (ASM61, clinical isolate M112 and 98BR004, respectively) in addition to H87Q and may be suspected to contribute to resistance. Mutations in this N-terminal loop have been reported to influence retroviral tropism and, therefore, may impact susceptibility to restriction factors [72]. The three CA loops—the CypA-binding region, the loop around P122 and the N-terminal loop—were reported to undergo concerted conformational shifts [73] and, therefore, may represent a cooperative functional unit. All other amino acid changes outside the CypA binding loop occurred in resistant, as well as in sensitive virus strains.

The amino acid polymorphism T54A, which is outside the CypA binding loop and was reported to modulate the sensitivity of HIV-1 to cyclosporines [74], was not found in our collection of naturally occurring DEB025 resistant virus strains.

### 2.7. Presumed Mode-of-Action of Cyclosporines in Inhibition of HIV-1

The precise mode of action of cyclosporines in inhibition of HIV-1 is not fully elucidated yet. DEB025 interaction with CypA blocks an essential function of CypA early in the viral replication cycle, which leads to impaired progression and/or completion of reverse transcription and, finally, to lack of transport of the pre-integration complex to the nucleus [41]. Removal of CA protein from the pre-integration complex was observed before entry of the complex to the nucleus [75]. For removal of CA from the pre-integration complex, stabilization of a certain conformation of the binding loop by CypA or a conformational change induced by the isomerase activity of CypA to the binding loop may be required. Inhibition of this “uncoating” step, by competitive binding of DEB025 to CypA, may allow the restriction factor Ref-1 to recognize CA and to abort productive HIV-1 infection by premature disassembly of the core.

Interestingly, HIV-1 variants being resistant to HIV-1 protease inhibitors often contain additional mutations in Gag noncleavage sites, in particular the H87Q/P polymorphism in CA [76]. This H87Q/P substitution appeared to increase viral fitness, since it conferred a replication advantage to the mutated virus, especially in CypA rich cell lines. Introduction of the H87Q change in viruses containing the P90A mutation, which is known to impair CypA binding, restored the defect in virus replication of HIV-1 P90A. Structural modeling of the CypA-binding loop revealed that the conformation of the loop containing P90A is highly distorted. H87Q introduction restored the conformation of the P90A containing loop, close to that of HIV-1 wild-type virus [76]. These results support the assumption that CypA-binding to CA stabilizes or induces a conformation necessary for successful virus replication and that the H87Q polymorphism stabilizes this conformation in the absence of CypA binding.

### 2.8. Presumed Mode-of-Action of Cyclosporines in Inhibition of HCV

DEB025 is also a potent inhibitor of HCV replication. The anti-HCV activity of DEB025 has been shown to be linked to its interaction with cyclophilins, in particular with CypA, which is a crucial cellular cofactor in HCV replication [8,17,50,51]. Though the mode of action seems to be different from that in HIV-1 replication—DEB025 is assumed to block CypA-mediated stimulation of the HCV RNA synthesis machinery—there may be similarities at the molecular level. Recently, Coelmont *et al*. [7] reported the isolation and characterization of DEB025 resistant HCV replicons only after long-term cell culture in the presence of the compound (20 weeks in average), indicating a high genetic barrier to resistance for DEB025. The only mutation consistently selected was mapped to NS5A, a gene coding for a protein involved in viral RNA synthesis, not to NS5B, the polymerase gene, and conferred a low level of resistance to DEB025 (approximately four-fold change in IC_50_). DEB025 resistant replicons replicated efficiently in CypA knockdown cells. Nevertheless, interaction between CypA and NS5A from DEB025 resistant replicons still occurred and could be blocked by DEB025. Thus, the mutation rendered the HCV replicon CypA-independent. NMR studies with peptides from NS5A containing the DEB025 resistance mutation revealed a difference to peptides from wild-type replicons regarding the relative amounts of two NS5A conformations. These data suggest that the resistance of HCV replicons to DEB025 may be caused by stabilization of a certain conformation of this NS5A region by the mutation, reducing the need for CypA-mediated stabilization and/or isomerization of this region. Thus, the mode of action of DEB025 inhibition of HIV-1 and HCV replication may be based on CypA-induced conformational changes or stabilization of certain conformations in crucial viral proteins.

**Table 2:** Capsid polymorphisms in HIV-1 isolates from DEB025 clinical trial and correlation with sensitivity to DEB025.

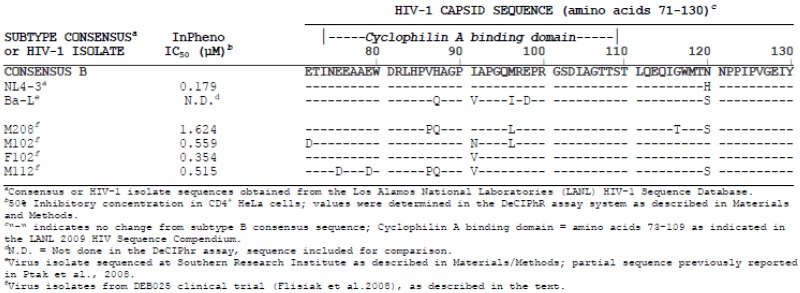

## 3. Experimental Section

DEB025 was synthesized using CsA as starting material based on the strategies described previously [36,38]. HIV-1 laboratory strains and clinical isolates were obtained from the NIAID Research and Reference Reagent Program (as given in [41]). The experimental procedures to determine antiviral activity were essentially the same as described in Ptak *et al*. for human PBMC [41] and in Chatterji *et al*. for TZM-bl cells [43]. In brief, CD4^+^ HeLa cells containing the reporter gene for beta-galactosidase (TZM-b1 cells) were infected with HIV-1 in the presence of various concentrations of DEB025. Infection was scored by X-gal staining and IC_50_ values were calculated by comparison with the untreated control. For the antiviral assay with PBMC, HIV-1 replication was quantified by supernatant reverse transcriptase activity 7 days post-infection. Sequences of the CA region of HIV-1 isolates were obtained from the Los Alamos National Laboratories (LANL) HIV-1 Sequence Database or determined at Southern Research Institute, as described in Ptak *et al*. [41]

HIV-1 containing serum was collected from the patients of the clinical study evaluating DEB025 in HIV-1/HCV-coinfected patients [47] at the day of the first visit (day-28). Evaluation of the activity of DEB025 against viruses carrying the capsid genes of HIV-1 from these patients was performed under contract by InPheno AG (Basel, Switzerland). To test the sensitivity of a patient’s virus to inhibition by DEB025, the viral CA protein gene was cloned into a proviral vector representing the viral background of NL4-3 (GenBank accession number #AF324493) to replace the NL4-3 CA protein gene. Recombinant chimeric fully infectious HIV-1 carrying the CA gene of virus isolated from each of the patients in the genetic background of HIV-1 strain NL4-3 was produced. The antiviral activity of DEB025 was determined in replicative HIV-1 infection of CD4^+^ HeLa cells containing the reporter gene for beta-galactosidase: “Principle of Dual-Enhancement of Cell-Infection to Phenotype Resistance (DeCIPhR)”, a proprietary assay system of InPheno AG. A range of twelve concentrations of DEB025 was tested in triplicates in the DeCIPhR assay system. The colorimetric read-out was translated into percentage of viral inhibition and processed by statistical curve fitting software. IC_50_ values were calculated from the inhibition curves using integral calculations. Sequence analysis of the CA region of the virus isolates from the four patients, who did not respond to DEB025 by a decrease in HIV-1 virus load, was also performed by InPheno AG under contract. Sequencing was performed using the ABI Big-Dye Terminator technology (Applied Biosystems, Foster City, Ca, USA), as per the manufacturer’s instructions, and reaction products were run on an ABI3100 prism sequencer.

CypA concentrations in PBMCs were measured via specific enzyme-linked immunosorbent assay (ELISA) using purified antibodies specific for CypA, as described in Flisiak *et al*. [47]

## 4. Conclusions

To conclude, two amino acid polymorphisms in the CA protein of HIV-1 could be clearly correlated with resistance to DEB025—H87Q and I91N. Both are located in the CypA-binding loop of CA. The H87Q change was by far more abundant than I91N. Additional polymorphisms in the CypA-binding loop (positions 86, 91 and 96), as well as in the N-terminal loop of CA may contribute to the degree of resistance. It is plausible to assume that these amino acid changes modulate the conformation of the CypA-binding loop of CA in such a way that binding and/or isomerase function of CypA is not needed anymore, thus rendering virus replication CypA-independent. This modified conformation may be such that the restriction factor Ref-1 cannot bind and/or exert its function.

The information about the polymorphisms leading to resistance of HIV-1 strains to DEB025 presented here may allow the identification of potential nonresponders in future clinical trials of DEB025 against HIV-1.

## Supplementary Files

Supplementary File 1 Supplementary Material (JPG, 3743 KB)
