# Supplementary figures and images for: Correlation of Naturally Occurring HIV-1 Resistance to DEB025 with Capsid Amino Acid Polymorphisms
Source: Viruses. 2013 Mar 22;5(3):981–97. doi: 10.3390/v5030981 (PMC3705307; doi:10.3390/v5030981)

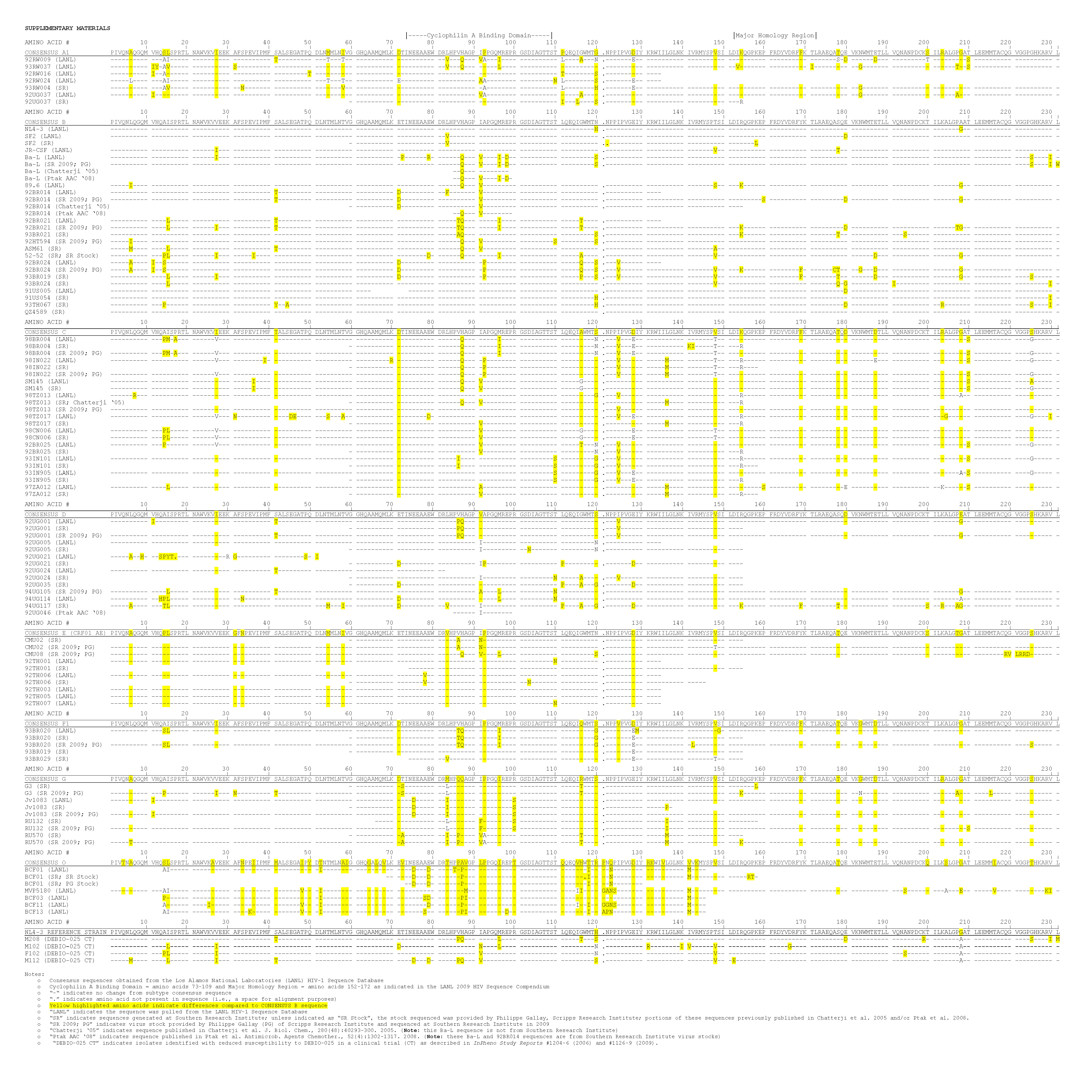

Supplement: Supplementary File 1 — Supplementary Material (JPG, 3743 KB) [file viruses-05-00981-s001.jpg]
